# Phenyllactic Acid Produced by *Geotrichum candidum* Reduces *Fusarium sporotrichioides* and *F. langsethiae* Growth and T-2 Toxin Concentration

**DOI:** 10.3390/toxins12040209

**Published:** 2020-03-26

**Authors:** Hiba Kawtharani, Selma Pascale Snini, Sorphea Heang, Jalloul Bouajila, Patricia Taillandier, Florence Mathieu, Sandra Beaufort

**Affiliations:** Laboratoire de Génie Chimique, UMR 5503, Université de Toulouse, CNRS, INPT, UPS, 31326 Toulouse, France; hiba.kawtharani@toulouse-inp.fr (H.K.); selma.snini@toulouse-inp.fr (S.P.S.); sorphea.itc@gmail.com (S.H.); jalloul.bouajila@univ-tlse3.fr (J.B.); patricia.taillandier@toulouse-inp.fr (P.T.)

**Keywords:** phenyllactic acid, biocontrol agent, T-2 toxin, *F. langsethiae*, *F. sporotrichioides*, G. candidum, mycotoxin.

## Abstract

*Fusariumsporotrichioides* and *F. langsethiae* are present in barley crops. Their toxic metabolites, mainly T-2 toxin, affect the quality and safety of raw material and final products such as beer. Therefore, it is crucial to reduce *Fusarium spp*. proliferation and T-2 toxin contamination during the brewing process. The addition of *Geotrichum candidum* has been previously demonstrated to reduce the proliferation of *Fusarium spp*. and the production of toxic metabolites, but the mechanism of action is still not known. Thus, this study focuses on the elucidation of the interaction mechanism between *G.* *candidum* and *Fusarium spp*. in order to improve this bioprocess. First, over a period of 168 h, the co-culture kinetics showed an almost 90% reduction in T-2 toxin concentration, starting at 24 h. Second, sequential cultures lead to a reduction in *Fusarium* growth and T-2 toxin concentration. Simultaneously, it was demonstrated that *G. candidum* produces phenyllactic acid (PLA) at the early stages of growth, which could potentially be responsible for the reduction in *Fusarium* growth and T-2 toxin concentration. To prove the PLA effect, *F. sporotrichioides* and *F.* *langsethiae* were cultivated in PLA supplemented medium. The expected results were achieved with 0.3 g/L of PLA. These promising results contribute to a better understanding of the bioprocess, allowing its optimization at an up-scaled industrial level.

## 1. Introduction

Beer is the most consumed alcoholic beverage worldwide and the third most popular drink overall after water and tea. In 2018, beer production in the European Union was estimated to be nearly 406,050 10^8^ L and its consumption was calculated to be around 370,092 10^8^ L [1]. Barley is the main ingredient in the brewing process and its quality directly influences the characteristics of the final product. However, barley crops can be contaminated by several fungal species belonging to *Aspergillus, Penicillium* and *Fusarium* genera [2]. The latter is the most prevalent genus all over the world and the main genus in Europe [3]. *Fusarium* species are responsible for the production of toxic metabolites called mycotoxins, which are of increasing concern at both health and economic levels [4]. Indeed, recent surveys carried out in Europe have demonstrated that barley crops are frequently contaminated by *Fusarium* species and their associated mycotoxins [5,6,7,8]. The use of such contaminated raw materials in the brewing process impacts the quality of the produced beer [9]. *Fusarium* species can produce several kinds of toxins belonging to the trichothecenes family, of which types A and B are commonly found in food and feed. The most important of them are deoxynivalenol (DON), nivalenol (NIV), T-2 and their derivatives: the 15-acetyldeoxynivalenol (15-ADON), 3-acetyldeoxynivalenol (3-ADON) and HT-2 toxin [10].

The T-2 toxin belonging to the type A family was first isolated in *F. tricinctum* cultures, now called *F. sporotrichioides* and then detected in several cereal grains such as wheat, oats, barley and their derivatives. T-2 toxin is mainly produced by *F. sporotrichioides* and *F. langsethiae* [11,12].

T-2 toxin is known to be the most cytotoxic of the type A trichothecenes and has adverse effects on cellular metabolism [13]. It is 1.5–1.7 times more toxic than its deacetylate form HT-2 toxin. Even though its carcinogenicity was proven in certain affected animals, no evidence of such effect was detected in humans. Therefore, the IARC classified the T2-toxin in group 3 as not classifiable with regard to its carcinogenicity for humans [14], thus leading the European Union (EU) to propose recommendations on the presence of T-2 toxin in cereals and cereal products. Thus, the maximum limits in unprocessed cereals are 100 µg/kg for wheat, rye and other cereals, 200 µg/kg for barley (including malting barley) and corn and 1000 µg/kg for oats. [15].

In order to limit mycotoxin contamination, several pre-harvest and/or post-harvest methods can be adopted [16,17,18]. These techniques either directly target fungal development or limit mycotoxin levels. Pre-harvest methods include good agricultural practices (GAPs) and good manufacturing practices. Crop rotation, tillage and fungicide treatment are mainly implemented to control fungal infection [19,20]. Fungicides are commonly used during agricultural practices but have numerous disadvantages such as detrimental effects on human and animal health, environmental contamination and subsequently, they have a strong impact on microbial biodiversity [21,22]. Indeed, fungicides of the azole family are used in small grain cereals to control *Fusarium spp.* They target the CYP51 (sterol 14α-demethylase) an important enzyme involved in ergosterol biosynthesis, which is essential to maintain fungal membrane fluidity and permeability [23]. By reducing fungal growth, they disturb the natural microbial ecosystem, causing the potential emergence of new microorganisms that may be even more dangerous [24]. Moreover, fungal resistance to these compounds has developed in recent years, thus reducing their effectiveness [25]. In an attempt to limit the proliferation of these toxinogenic and phytopathogens fungal species, biocontrol approaches are starting to be published. Recently, Rahman et al. (2018) proposed the concept of the “plant holobiont”. They demonstrated that barley is consistently associated with beneficial bacteria inside their seeds and that this type of association should be encouraged to help the plant react to fungal attack. This could open up new possibilities for applying seeds formulated with endophytic bacteria as bioinoculants for sustainable agriculture [26]. Post-harvest methods include physical treatments such as high temperature treatment exposure and chemical agents. However, these procedures can lead to the deterioration of nutritional quality and alteration of the organoleptic properties of the food matrix [27,28,29]. Therefore, it is important to conceive a bioprocess to minimize these side effects. This implies the use of natural and environmentally friendly ways to maintain the safety and the quality of the final product. The brewing process comprises several stages and among them, the malting step provides the best conditions (22 °C and high humidity) for *Fusarium* development and T-2 toxin production [30,31]. To reduce mycotoxin concentration during the malting process, several studies have reported the use of lactic acid bacteria (LAB), which are characterized by their antifungal and anti-mycotoxigenic properties [32,33]. However, LAB are fermenting bacteria and can spoil beer, leading to acidification, turbidity, off-flavors and ropiness, depending on the bacterial strain [34,35].

The French Institute for Brewing and Malting (IFBM) filed a patent in September 1999 entitled “The inoculation by *Geotrichum candidum* during malting of cereals or other plants” [36]. The invention consists of using *G. candidum* strain, a filamentous yeast, to inhibit the development of undesirable microorganisms such as *Fusarium spp.* during the malting process to avoid the contamination of beer products by T-2 toxin. Antibacterial activity was previously attributed to this microorganism as it can inhibit the growth of several bacteria such as *Listeria monocytogenes* [37]. *G. candidum* was also found to inhibit other Gram-positive bacteria, such as *Staphylococcus aureus* and *Enterococcus faecalis*, and Gram-negative bacteria, such as *Providencia stuartii* and *Klebsiella oxytoca* [38]. As a matter of fact, three metabolites produced by *G. candidum* have been reported as antimicrobial compounds. Phenyllactic acid (PLA) and indoleacetic acid (ILA) induce behavioral and structural alterations to *L. monocytogenes,* which completely inhibit its growth [37]. The third metabolite, phenylethyl alcohol (PEA), is responsible for the “aromatic rose” character of soft cheese, and promotes membrane damage and inhibition of RNA and protein synthesis of Gram-positive and Gram-negative bacteria, such as *S. aureus* and *Escherichia coli* [39]. Among these three metabolites, PLA is the most effective against bacteria growth [37].

However, the *G. candidum* mechanism against *Fusarium spp.* and T-2 toxin production during the malting process is still unidentified. Given the data in the literature considering PLA as a powerful antimicrobial, the production of PLA by *G. candidum* now needs to be monitored and its effect on *Fusarium spp*. growth as well as on T-2 toxin concentration needs to be quantified.

Thus, this study aims to decipher the interaction mechanisms between *G. candidum* and two *Fusarium* strains: *F. langsethiae* 2297 and *F. sporotrichioides* 186, determine on which level these interactions occur and identify the metabolite responsible for the T-2 toxin concentration reduction.

## 2. Results

### 2.1. Effect of Co-Culture between G. candidum and Fusarium Strains on Fungal Growth and T-2 Toxin Concentration

The co-culture experiment consisted of simultaneously inoculating *G. candidum* and *Fusarium* strains into Ym medium for different incubation times (ranging from 24 to 168 h) at 22 °C, 150 rpm. For each incubation time, microbial dry weight, T-2 toxin and PLA concentrations were analyzed in control cultures (*G. candidum, F. langsethiae* 2297 *and F. sporotrichioides* 186 alone) and in co-cultures. Two co-culture experiments were conducted: *G. candidum* with *F. langsethiae* 2297 (Gc/Fl) and *G. candidum* with *F. sporotrichioides* 186 (Gc/Fs).

**Figure 1:**
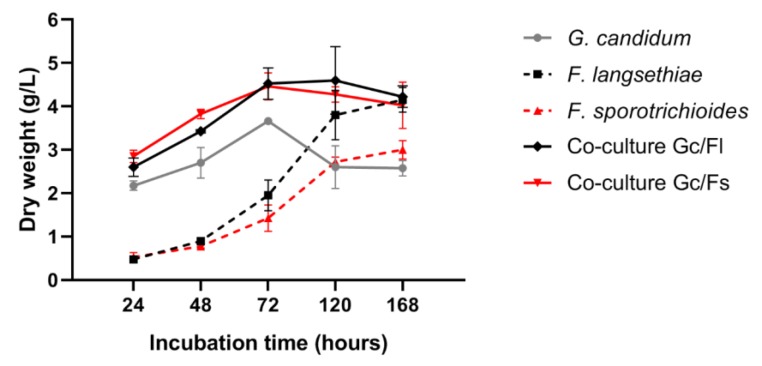
Microbial dry weight analysis in control cultures (*G. candidum,* *F. langsethiae* 2297 and *F. sporotrichioides* 186 alone) and in co-culture experiments (*G. candidum* with *F. langsethiae* 2297 and *G. candidum* with *F. sporotrichioides* 186).

In control cultures, *G. candidum* dry weight increased during the first 3 days of incubation reaching 3.9 g/L and then slightly decreased to stagnate at 2.3 g/L during the last hours of the experiments. For *Fusarium* control cultures, fungal biomass increased throughout the whole experimental duration; *F. langsethiae* 2297 attained a maximum of 3.8 g/L whereas *F. sporotrichioides* 186 almost reached 3 g/L. In co-culture conditions, where microorganisms were simultaneously inoculated, for the two co-culture experiments (Gc/Fl and Gc/Fs), the total biomass increased during the first 3 days of incubation and then stabilized until the end of the experiment. However, in both co-culture experiments, for each incubation time, the total microbial dry weight was not the sum of dry weights obtained separately in control culture. Thus, co-culture leads to microbial growth reduction without distinguishing the growth of *G. candidum* from *Fusarium* species (Figure 1).

**Figure 2:**
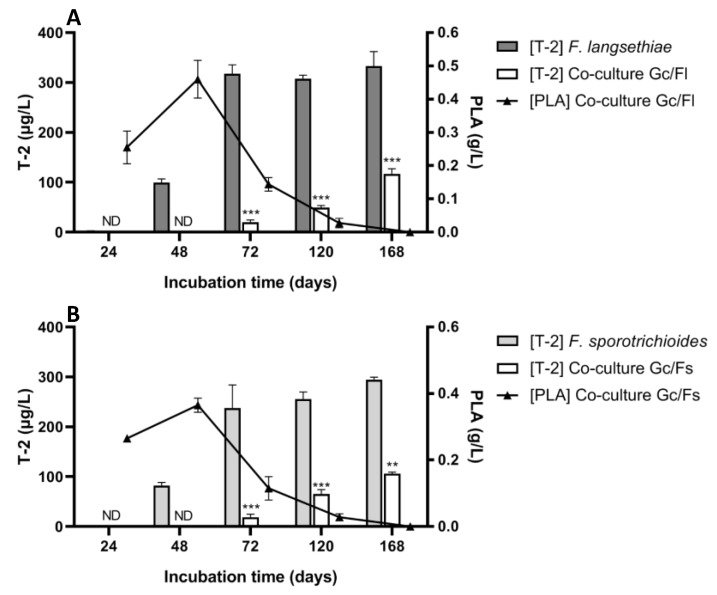
T-2 concentration (µg/L) and phenyllactic acid (PLA) concentration (g/L) in co-culture experiments. Panel **A**: Co-culture experiment of *G. candidum* and *F. langsethiae* 2297. Panel **B**: Co-culture experiment of *G. candidum* and *F. sporotrichioides* 186 (One-way ANOVA, Tukey’s multiple comparisons post-hoc test, ** *p*-value < 0.01; *** *p*-value < 0.001) ND = not detectable.

Figure 2, Panel A, shows that in control culture (*F. langsethiae* 2297 alone), T-2 toxin was detected from 48 h and the concentration was 99.65 µg/L (±7.28), and reached 332.7 µg/L (±29.42) after an incubation time of 168 h. In co-culture (*G. candidum* with *F. langsethiae* 2297), T-2 toxin was detected from 72 h (20.22 µg/L ± 4.32) and attained 116.44 µg/L (±10.89) after incubation for 168 h. The percentage of T-2 toxin reduction was 100%, 94%, 84% and 65% at 48 h, 72 h, 120 h and 168 h, respectively. These results were similar to those previously obtained for the *F. langsethiae 033* strain [40]. The same phenomenon was observed in the second co-culture experiment using *F. sporotrichioides* 186 strain with slightly different degrees of reduction (Figure 2, Panel B). In control culture (*F. sporotrichioides* 186 alone), T-2 toxin was detected from 48 h and the concentration was 82.3 µg/L (±6.1), reaching 294.65 µg/L (±4.74) after incubation for 168 h. As for the first co-culture experiment, in the co-culture *G. candidum* with *F. sporotrichioides* 186, T-2 toxin was detected from 72 h (18.8 µg/L ± 6.12) and reached 106.25 µg/L (±3.04) after incubation for 168 h. The percentage of T-2 toxin reduction was 100%, 92%, 74% and 64% at 48 h, 72 h, 120 h and 168 h, respectively. To ensure that T-2 toxin was not degraded, HT-2 toxin was also monitored and was not detected.

In both co-culture experiments, the PLA concentration increased rapidly during the first two days of incubation. In the co-culture with *F. langsethiae* 2297, PLA concentration attained 0.25 g/L (±0.05) at 24 h and 0.46 g/L (±0.06) at 48 h. Afterward, it radically decreased starting at 72 h (0.14 g/L± 0.02) to reach a null value at the end of the incubation time. The same profile was observed in the co-culture with *F. sporotrichioides* 186: PLA concentration attained 0.26 g/L (±0.02) at 24 h and 0.36 g/L (±0.04) at 48 h. PLA concentration in co-culture conditions was inversely proportionate to the T-2 toxin concentration. Indeed, the increase in T-2 toxin concentration was correlated with the reduction of PLA concentration in the medium. When PLA was at its highest level (0.46 g/L in Gc/Fl and 0.36 g/L in Gc/Fs), T-2 toxin was not detected.

### 2.2. G. candidum Growth and PLA Production Kinetics

The *G. candidum* strain selected by the IFBM and used in this study produces PLA during the brewing process. To study the growth of this filamentous yeast, Ym medium was initially inoculated with 0.2 g/L of a *G. candidum* starter culture and incubated at 22 °C, 200 rpm for 5 days. Samples were withdrawn at the starting point and after 6 h, 12 h 24 h, 48 h, 72 h, 96 h, and 120 h of fermentation time and the PLA concentrations were measured.

**Figure 3:**
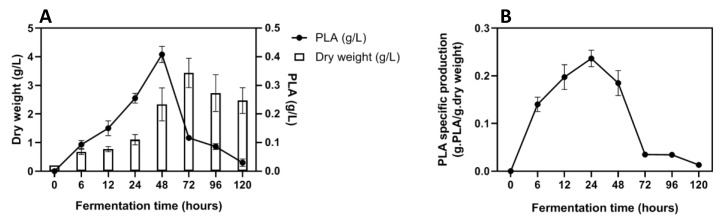
PLA concentration (g/L) and *G. candidum* biomass (g/L) in Ym medium (Panel **A**) and PLA specific production (g PLA/g dry weight) in Ym medium (Panel **B**).

After 48 h of incubation, the concentration of PLA reached a maximal concentration of 0.41 g/L for 2.25 g/L of yeast dry weight. After 72 h of culture, both *G. candidum* dry weight and PLA concentration started decreasing, growth went from a maximum of 3.43 g/L (±0.51) to 2.46 g/L (±0.46) and PLA concentration drastically decreased from a maximum of 0.41 g/L (±0.03) to 0.03 g/L (±0.01) (almost 17 times less) (Figure 3, Panel A). Figure 3, Panel B demonstrates the specific production of PLA relative to *G. candidum* biomass through the fermentation time. It clearly shows that the PLA is highly accumulated in the medium at the early stages of *G. candidum* growth between 12 and 48 h and then drastically disappears.

### 2.3. Sequential Cultures

This experiment studied the indirect interactions between *G. candidum* and the two *Fusarium* strains. Therefore, the same Ym medium used in Section 2.2 to grow *G. candidum* was filtrated after 6 h, 12 h, 24 h, 48 h, 72 h, 96 h and 120 h of fermentation time into sterilized Erlenmeyer flasks. Henceforth, the obtained filtrate will be called the “pre-fermented medium” which contains all the metabolites secreted by *G. candidum*.

**Figure 4:**
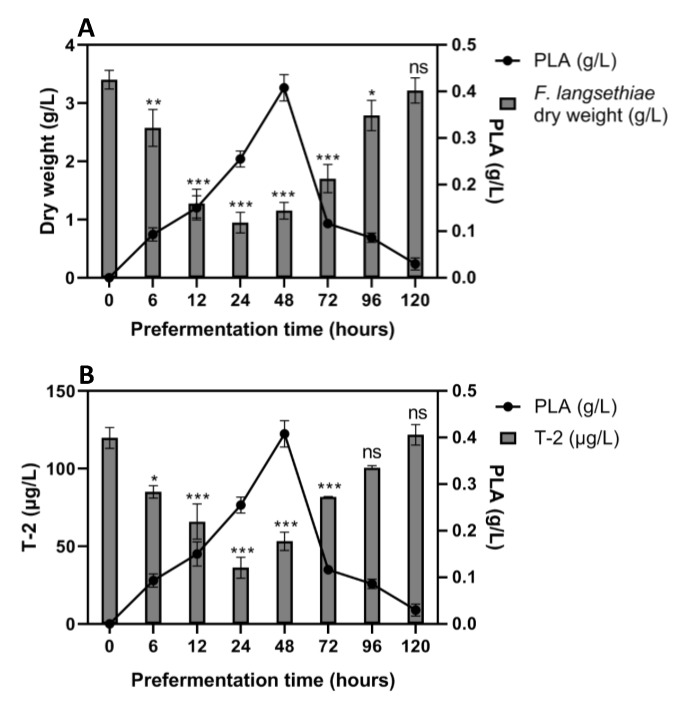
Sequential culture of *F. langsethiae* 2297 inoculated in pre-fermented medium by *G. candidum* and incubated 7 days at 22 °C. Panel **A**: Dry weight of *F. langsethiae* 2297 (g/L) in comparison with PLA concentration (g/L). Panel **B**: T-2 concentration (µg/L) in comparison with PLA concentration (g/L). One-way ANOVA, Dunnett multiple comparisons post-hoc test, * *p*-value < 0.05; ** *p*-value < 0.01; *** *p*-value < 0.001; ns = not significant).

In the sequential culture experiment with *F. langsethiae* 2297, the dry fungal weight was gradually reduced on pre-fermented media up to 48 h (Figure 4, Panel A). The most significant reduction in the *F. langsethiae* 2297 dry weight occurred in the flasks pre-fermented for 12 h, 24 h and 48 h with a 62%, 72% and 66% reduction percentage, respectively. Beyond 24 h of pre-fermentation, it appeared that *F. langsethiae* 2297 growth increased slowly. In Ym medium pre-fermented for 120 h, the fungal strain was able to proliferate naturally (3.1 g/L of fungal biomass compared to 3.4 g/L in a non-fermented Ym medium). These results demonstrated that the fungal growth inhibition was more efficient in Ym medium pre-fermented for two days by *G. candidum*. Previous results showed that the PLA was produced during the early growth phase of the yeast reaching its peak (between 0.25 and 0.41 g/L of PLA) at around 24–48 h of fermentation time. This suggests that the PLA was involved in the reduction of fungal biomass at a rate of 72% (going from 3.4 g/L in a non-fermented medium to 0.95 g/L in 24 h pre-fermented medium). A significant reduction in T-2 toxin concentration was observed for fungal cultures performed in Ym medium pre-fermented from 6 h to 72 h (Figure 4-Panel B). To ensure that T-2 toxin was not degraded, HT-2 toxin was also monitored and was not detected.

The most significant reduction in T-2 toxin concentration occurred in the flasks pre-fermented for 24 h and 48 h, with a 70% and 56% reduction, respectively. These percentages correlated perfectly with the biomass reduction rate (72% and 66%, respectively). This suggested that the reduction in fungal biomass in the medium is responsible for the reduction in T-2 toxin concentrations. Indeed, specific productions were calculated and demonstrated that the T-2 toxin reduction is correlated to fungal biomass reduction (data not shown).

**Figure 5:**
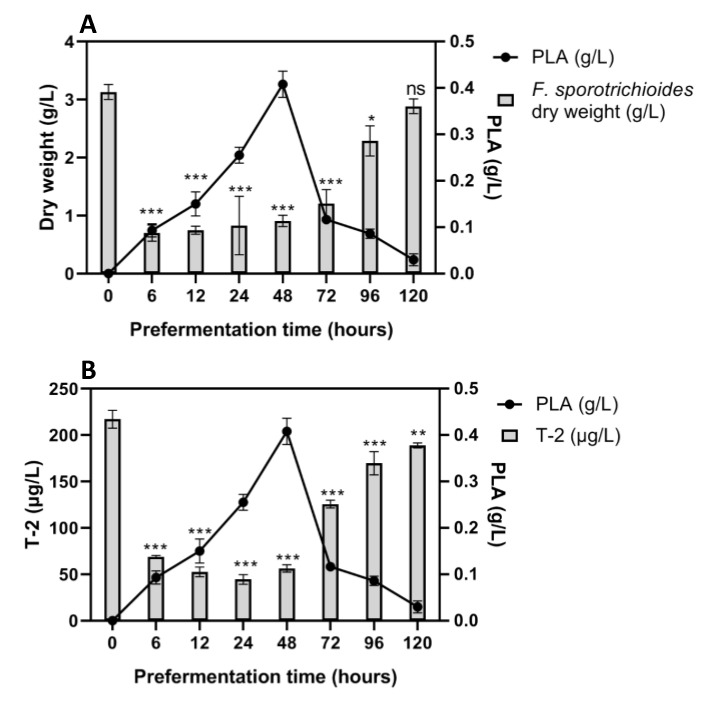
Sequential culture of *F. sporotrichioides* 186 inoculated in pre-fermented medium by *G. candidum* and incubated for 7 days at 22 °C. Panel **A**: Dry weight of *F. sporotrichioides* 186 (g/L) in comparison with PLA concentration (g/L). Panel **B**: T-2 concentration (µg/L) in comparison with PLA concentration (g/L). One-way ANOVA, Dunnett multiple comparisons post-hoc test, * *p*-value < 0.05; ** *p*-value < 0.01; *** *p*-value < 0.001; ns = not significant).

The same experiment was conducted using *F. sporotrichioides* 186 (Figure 5). Fungal growth was drastically reduced in medium pre-fermented for 6 h, 12 h, 24 h and 48 h at almost the same rate of 70% in correlation with the increase of PLA in the medium. As expected, the Ym medium pre-fermented for 6 h, 12 h, 24 h and 48 h showed an important reduction in T-2 toxin of up to 78%. The equivalence between the growth reduction and the toxin reduction percentages also suggests that it is due to the cessation of fungal growth.

These experiments demonstrated that the interaction between *G. candidum* and *Fusarium* strains occurs through a compound released by *G. candidum* in the medium. As previous results showed, it is highly probable that the PLA, present in *G. candidum* filtrate is the metabolite responsible for the reduction of fungal dry weight and the subsequent reduction in T-2 toxin concentration. To validate this hypothesis, further experiments using pure PLA compound were required.

### 2.4. Effect of Pure PLA on Fungal Growth and T-2 Toxin Concentration

D-(+)-3Phenyllactic acid was purchased as a pure compound and several concentrations were tested. To validate the results presented in previous sections, PLA solution was prepared at concentrations found at different fermentation times: 0.5 g/L, 0.4 g/L, 0.3 g/L and 0.2 g/L. Lower concentrations of PLA were also tested to determine the minimal inhibitory concentration (MIC): 0.05 g/L and 0.1 g/L. The effect of this pure compound on *Fusarium* strains growth and its ability to produce T-2 toxin was evaluated.

**Figure 6:**
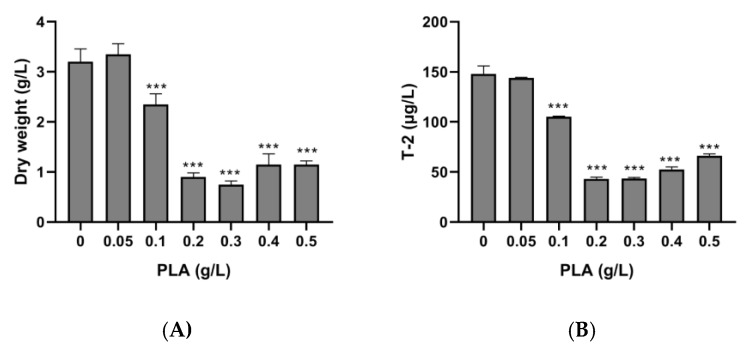
Effect of PLA on the dry weight of *F. langsethiae* 2297 (**A**) and T-2 toxin concentration (**B**) (One-way ANOVA, Dunnett multiple comparisons post-hoc test, *** *p*-value < 0.001).

Both *F. langsethiae* 2297 growth and T-2 toxin production were highly affected by the addition of D-PLA in Ym medium (Figure 6). As the concentration of D-PLA increased in the medium, lower fungal mass and lower toxin concentration were quantified. In the control condition (without PLA) *F. langsethiae* 2297 dry weight was 3.2 g/L (±0.26) and the T-2 toxin concentration was 148 µg/L (±7.8), whereas in the presence of 0.3 g/L of PLA, both dry weight and T-2 concentration were reduced to a rate of 71%, reaching 0.75 g/L (±0.7) and 43.4 µg/L (±1.2), respectively.

**Figure 7:**
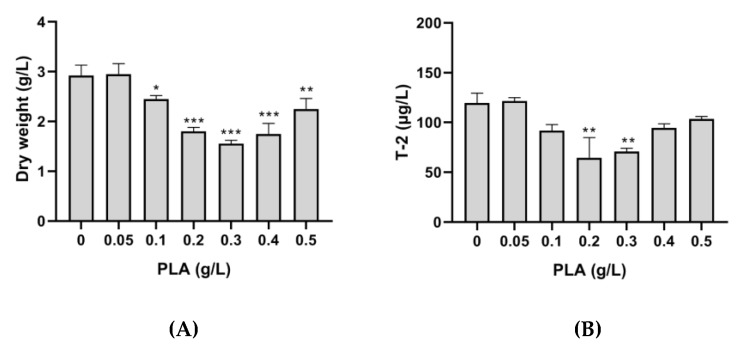
Effect of phenyllactic acid (PLA) on dry weight of *F. sporotrichioides* 186 (**A**) and T-2 toxin concentration (**B**) (One-way ANOVA, Dunnett multiple comparisons post-hoc test, * *p*-value < 0.05; ** *p*-value < 0.01; *** *p*-value < 0.001).

The same PLA concentrations were tested on *F. sporotrichioides* 186 and similar results were obtained (Figure 7). The most important reduction occurred in Ym medium supplemented with 0.3 g/L of D-PLA.

**Figure 8:**
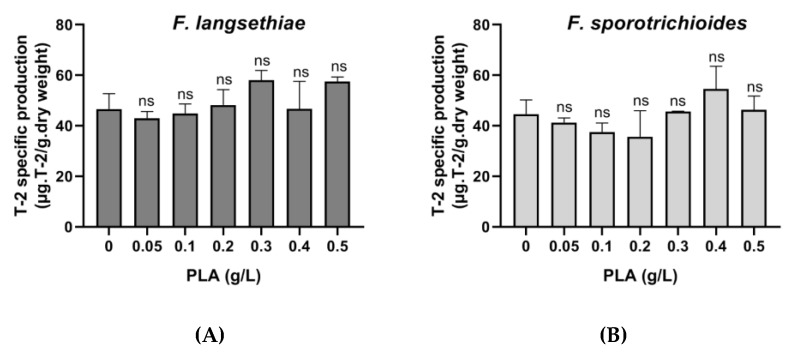
Specific production of T-2 toxin by *F. langsethiae* 2297 (**A**) and *F. sporotrichioides* 186 in Ym medium supplemented with pure phenyllactic acid (PLA) (**B**) and incubated 7 days at 22 °C (One-way ANOVA, Dunnett multiple comparisons post-hoc test, ns = not significant).

In both cases, it seems clear that the reduction of T-2 toxin concentration in the medium is directly related to the reduction in fungal growth. Specific production was calculated for each PLA concentration and demonstrated that the T-2 toxin reduction is correlated to the fungal biomass reduction (Figure 8).

## 3. Discussion

**Figure 9:**
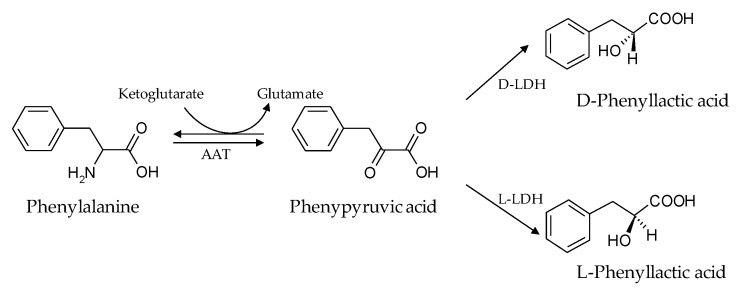
Hypothetical phenyllactic acid biosynthesis pathway. Adapted from Chaudhari and Gokhale (2016) [47]. AAT: amino acid transferase; D-LDH: D-lactate dehydrogenase; L-LDH: L-lactate dehydrogenase.

The contamination of food raw material by fungal species has many consequences. In addition to the alteration of commodities, the loss of nutritional qualities, and the strong reduction in yield, fungal development can lead to the accumulation of toxic compounds such as mycotoxins. In France, the occurrence of several *Fusarium* species in barley crops intended for brewing has become a source of concern over the past ten years. In barley crops, the introduction of *F. sporotrichioides* and *F. langsethiae* has been recently observed, progressively replacing *F. poae* [41,42,43]. The risk associated with these *Fusarium* species is the production of T-2 toxin, the most toxic compound in the type-A trichothecenes family. During the brewing process, the malting step provides the best conditions (22 °C and high humidity) for *Fusarium* development and T-2 toxin production [30]. Currently, *G. candidum* is used during the brewing process to reduce T-2 toxin contamination. However, its efficiency is variable and the mechanisms of interaction between *G. candidum* and *Fusarium* species are still unknown. Previously, Gastélum-Martinez et al. (2012) used the co-culture method between those two microorganisms and demonstrated that the direct interaction between *G. candidum* and *F. langsethiae* 033 led to a drastic T-2 toxin concentration reduction (93% in comparison to the control culture) [40]. To decipher the mechanism of interaction that lead to this reduction, in the presented study, the two microorganisms were also cultivated sequentially. First, *G. candidum* was cultivated, and removed from the medium before *Fusarium* strain inoculation. Several incubation times for *G. candidum* culture were tested (0 to 146 h) and *Fusarium* incubation was always 7 days. Results obtained in these sequential cultures show a reduction in the T-2 toxin concentration linked to a reduction in fungal growth. In addition, the reduction in T-2 toxin concentration varies according to the medium pre-fermentation time by *G. candidum*. In sequential cultures, T-2 toxin concentrations are inversely correlated with the production of PLA by *G. candidum,* demonstrating that the mechanism leading to T-2 toxin reduction, was linked directly to the PLA concentrations in the medium. Indeed, while the PLA concentration was at its highest level after 48 h of pre-fermentation time, the T-2 toxin concentration was at its lowest. The correlation between *G. candidum* growth evolution and PLA concentration in the medium suggests that PLA is a primary metabolite as it is secreted during the growth phase (from 0 h to 48 h, the PLA concentration varied from 0 to 0.41 g/L) and then gradually disappeared from the culture media. PLA biosynthesis is not yet described in *G. candidum* but well described in lactic acid bacteria (LAB) strains, which can produce large amounts of PLA. In fact, in lactic acid bacteria, the PLA results from amino acid metabolism of phenylalanine and α-ketoglutarate. In a glucose, citric acid or fructose enriched medium, the phenylalanine amino acid group is transferred to α-ketoglutarate under the action of aromatic amino acid transferase (AAT), leading to the formation of phenylpyruvic acid (PPA), an intermediate to PLA. Depending on the type of lactate dehydrogenases (L-LDH or D-LDH) present in lactic acid bacteria, PPA is converted to either L-PLA or D-PLA [44,45,46]. A potential PLA synthesis pathway is explicitly demonstrated by Chaudhari and Gokhale (2016) and simplified in Figure 9 [47]. Studies have shown that the D form of PLA is more effective as an antimicrobial compound than the L form [37].

Several studies have been conducted on LAB and more precisely, on the *Lactobacillus* genus, which is frequently involved in their antifungal activity [48,49,50]. *Lactobacillus* strains and *L. plantarum* in particular, have been found to produce PLA in sourdough bread. The use of these strains is a means of natural food preservation. Indeed, studies have shown that they improve the shelf life of bread and bakery products by decreasing and/or inhibiting fungal activities. PLA is considered one of the responsible inhibitory compounds along with lactic acid and acetic acid [51]. To our knowledge, no studies have been carried out on PLA metabolism and its toxicity effect in the human body. In 2002, Lavermicocca et al. (2003) studied the fungicidal activity of PLA on 23 fungal strains belonging to *Aspergillus*, *Penicillium* and *Fusarium* genera. Among these strains, 90% showed at least a 50% growth inhibition at PLA concentrations lower than 7.5 g/L. Other strains presented a growth delay of at least three days [52]. Dieuleveux et al. have proved that PLA produced by *G. candidum* strains at 20 g/L also has antibacterial activity against *L. monocytogenes*, *S. aureus*, *E. coli* and *A. hydrophila* [37,38,53].

*Fusarium* strains used in this study were more susceptible to PLA that those tested by Lavermicocca et al. (2003). Indeed, *F. langsethiae* 2297 growth was drastically reduced (72%) when it was exposed to 0.2 g/L of PLA, whereas *F. sporotrichioides* 186 growth was slightly reduced (47%) when it was exposed to 0.3 g/L of PLA. Thus, there is relevant variability in susceptibility among fungal species. Although the antimicrobial action mechanism is still not elucidated, some suggest that the PLA causes the bacteria to form aggregates with the secretion of polysaccharides described as a “response to the attack”. Indeed, as the concentration of PLA increased, a larger amount of polysaccharides were found in the medium and alteration in cell wall rigidity after only 27 h of incubation was observed leading to cell death [38,47]. In this study, as indicated by the specific production of T-2 toxin obtained for each fungal strain, the reduction in the concentration of T-2 toxin is correlated with the reduction in fungal growth. However, in some cases, the inhibition of fungal growth by sub-lethal concentrations of fungicide or some natural products enhances mycotoxin production [54,55,56]. This must be taken into account in the development of biocontrol strategies.

In this study, to provide an explanation for the phenomenon of T-2 toxin concentration control during the malting process previously observed by the IFBM, in vitro experiments were carried out under environmental conditions close to those of the brewing process. Currently, the filamentous yeast is added in a freeze-dried form (100 g per 25 tons of barley) directly into the barley steeping water for at least 10 h. Then, the water is discarded and the steeped barley remains at rest for 3 to 5 days at 16–20 °C. This stage is the most critical step in the brewing process because the operating conditions favor *Fusarium* growth and T-2 toxin production. Results demonstrate that the reduction in *Fusarium* contamination and T-2 toxin during the malting process is due to the PLA produced by *G. candidum*. Based on the results of this study, in order to develop an effective biocontrol method to use *G. candidum*, preparation of the strain seems essential to activate the PLA production metabolism. Mu et al. developed a medium favorable to PLA production by *Lactobacillus sp.* strains, highly enriched with glucose, phenylpyruvic acid (phenylalanine intermediate in the PLA biosynthesis pathway) and yeast extract [46]. This medium significantly enhanced *Lactobacillus sp.* proliferation, and thus PLA yield. However, the use of such broth on an industrial level does not seem to be applicable for several reasons. On one hand, it may alter the organoleptic characteristics of the final product. On the other, using these components in large amounts would have a considerable economic impact on the industry. Consequently, it seems important to combine optimized growth factors (*G. candidum* activation medium and initial concentration, fermentation duration, temperature, water activity, rotation speed, oxygenation levels, etc.) to enhance PLA production naturally, and to develop an ecofriendly, toxin-free beer product. Moreover, the presence of PLA during the malting step not only helps to reduce *Fusarium* flora and consequently, to reduce T-2 toxin concentration, but it also improves the organoleptic properties of the final beer product [36,57,58]. This study demonstrates for the first time, the role of PLA as a biocontrol agent in reducing T-2 toxin concentration.

## 4. Materials and Methods

### 4.1. Reagents and Chemicals

T-2 toxin and phenyllactic acid (PLA) were purchased from Sigma-Aldrich (Saint-Quentin-Fallavier, France). Stock solutions were prepared in dimethylsulfoxyd (DMSO) and acetonitrile–water (30:70 v/v) mixture, respectively, and stored at −18 °C until use. Solvents used for T-2 toxin extraction and high-performance liquid chromatography (HPLC) were analytical grade quality and purchased from Thermo-Fisher Scientific (Illkirch, France). Ultrapure water used for HPLC was purified at 0.22 µm by an ELGA purification system (ELGA LabWater, High Wycombe, United Kingdom).

### 4.2. Strains, Media and Culture Conditions

In this study, two *Fusarium* strains were used: *F. sporotrichioides* 186 and *F. langsethiae* 2297. Both strains were previously isolated from contaminated barley kernels and were kindly provided by the French Institute of Brewing and Malting (IFBM). The filamentous yeast *Geotrichum candidum* is already used as a biocontrol agent during the malting process (IFBM Malting Yeast^®,^ DMS food specialties, La Ferté sous Jouarre, France) and was purchased from DSM Food Specialties.

*Fusarium* pre-cultures were performed on potato dextrose agar medium (PDA 39 g/L) and incubated at 22 °C for 7 days. Cultures were then used to induce sporulation or conserved at 4 °C. *Fusarium* strains sporulation was induced in carboxymethylcellulose (CMC) liquid medium (CMC: carboxymethylcellulose 15 g/L; yeast extract 1 g/L; MgSO_4_ 7H_2_O 0.5 g/L; NH_4_NO_3_ 1g/L; KH_2_PO_4_ 1 g/L). Briefly, at least 15 plugs of each *Fusarium* strain from a seven-day-old solid pre-culture were inoculated in 150 mL of CMC medium and incubated in an orbital shaker set at 22 °C at 150 rpm for 15 days in the dark. At the end of the incubation time, the solution was filtrated using sterilized Mira cloth. Spores were counted on Thoma cell counting chamber and ultimately used to inoculate culture during further experiments or conserved in 40% glycerol at −80 °C.

*G. candidum* strain was supplied in freeze-dried form, thus a pre-culture was essential to revivify it prior to experimental use. A 24 g/L culture was prepared in 250 mL of yeast and malt (Ym) liquid medium (Ym: glucose 5 g/L; yeast extract 1.5 g/L; malt extract 1.5 g/L; peptone salt 2.5 g/L pH 7) and incubated in an orbital shaker set at 22 °C at 150 rpm for 24 h. At the end of the incubation time, this culture was used as a starter culture.

Ym liquid medium was used during all experiments (co-cultures and sequential cultures) to elucidate the interaction mechanisms between *G. candidum* and *Fusarium* strains.

### 4.3. Kinetic of PLA Production by G. candidum

In an Erlenmeyer flask, 150 mL of Ym medium was inoculated with *G. candidum* starter culture with a final concentration adjusted at 0.2 g/L and then incubated in an orbital shaker set at 22 °C at 150 rpm for different fermentation times ranging from 6 h to 120 h. At the end of the fermentation time, the medium was aseptically divided into two volumes. First, 50 mL were used to evaluate *G. candidum* growth by measuring the dry weight and PLA concentration by HPLC-DAD at each sampling time. The remaining 100 mL was aseptically filtered to eliminate *G. candidum* cells, leaving only its excreted metabolites in the medium. The medium nutrients were then adjusted according to the volume and the pH was adjusted at 7. These volumes were used during the sequential cultures experiments and are henceforth referred to as pre-fermented medium. Experiments were conducted four times in triplicate.

### 4.4. Co-Culture of Fusarium Strains and G.candidum

Erlenmeyer flasks containing 150 mL of Ym medium were inoculated with *G. candidum* starter culture at the final concentration of 0.2 g/L. Then, *F. langsethiae* 2297 or *F. sporotrichioides* 186 was inoculated at a final concentration of 10^6^ spores/mL in their respective flasks. For control conditions, each microorganism was inoculated alone at the same concentrations. Cultures were incubated in an orbital shaker set at 22 °C at 150 rpm. Several incubation times were tested: 24 h, 48 h, 72 h, 120 h and 168 h. At the end of all sampling times for all culture conditions the total dry weight, PLA and T-2 toxin concentration were evaluated. All experiments were conducted twice in duplicate.

### 4.5. Sequential Cultures of Fusarium Strains and G.candidum

For sequential cultures, 100 mL of pre-fermented Ym medium at different fermentation times ranging from 6 h to 120 h (used in Section 4.3) were inoculated with *F. langsethiae* 2297 or *F. sporotrichioides* 186 at the final concentration of 10^6^ spores/mL in their respective flasks. Cultures were incubated in an orbital shaker set at 22 °C at 150 rpm for 7 days. For the control condition, *F. sporotrichioides* 186 or *F. langsethiae* 2297 were inoculated in a non-fermented Ym liquid medium at the same concentrations. At the end of the incubation time, fungal growth was evaluated by measuring the dry weight and T-2 toxin concentration by HPLC-DAD. All experiments were conducted twice in duplicates.

### 4.6. Phenyllactic Acid Effect on F. sporotrichioides 186 and F. langsethiae 2297 Growth and T-2 Toxin Concentration

To confirm that PLA is the metabolite produced by *G. candidum,* which is involved in *Fusarium* growth reduction and T-2 toxin concentration reduction, fungal cultures were conducted in Ym liquid medium supplemented with PLA. PLA standard stock solution was prepared at 40 mg/mL in a mixture of acetonitrile/water (30/70, v/v) and appropriate volumes of PLA stock solution were added in order to obtain several different concentrations: 0.05 g/L; 0.1 g/L; 0.2 g/L; 0.3 g/L; 0.4 g/L and 0.5 g/L in Erlenmeyer flasks containing 100 mL of Ym liquid medium. Then, *F. langsethiae* 2297 or *F. sporotrichioides* 186 was inoculated at the final concentration of 10^6^ spores/mL in their respective flasks. Cultures were incubated in an orbital shaker set at 22 °C at 150 rpm for 7 days. At the end of the incubation time, *Fusarium* strains’ growth was evaluated by measuring the dry weight and T-2 toxin concentration by HPLC-DAD. PLA dilutions were prepared to add only 75 µL of acetonitrile in the culture medium, this concentration having been identified as a no-effect dose on both fungal growth and T-2 toxin concentration. Control cultures were performed by adding only 75 µL of acetonitrile to the medium.

### 4.7. G. candidum, F. sporotrichioides 186 and F. langsethiae 2297 Biomass Evaluation

To estimate microorganism growth during the incubation period, vacuum filtration was performed to determine the dry weight (g/L). First, cellulose nitrate filters (pore size 0.45 µm, Sartorius Stedim Biotech, Goettingen, Germany) were left to dry overnight in an oven set at 105 °C. Afterward, 10 mL of culture medium were vacuum-filtered at each sampling time and filters were then incubated at 105 °C for 24 h. The microorganism dry weight refers to the difference between filters post-filtration and pre-filtration.

### 4.8. PLA and T-2 Toxin Quantification by HPLC-DAD

#### 4.8.1. PLA Quantification

At each sampling time, 1 mL of culture media was withdrawn and filtrated through 0.45 µm PTFE syringe filters (Thermo Scientific Fisher, Villebon-Sur-Yvette, France) to eliminate microorganisms from the supernatant prior to injection into HPLC apparatus. Analyses of PLA were performed using a Luna C18(2) column (5 µm, 250 × 4.6 mm) and a pre-column with the same characteristics (Phenomenex, Torrance, CA, USA). The detection of PLA was performed using a Dionex Ultimate 3000 UHPLC system coupled with a diode-array detector (DAD) set at 210 nm (Thermo Fisher Scientific, Illkirch, France). The analysis was performed in a gradient mode using acidified water (0.2% of acetic acid glacial) as solvent A and pure HPLC grade acetonitrile as solvent B. Flow was set at 1.2 mL/min with A/B ratios of 90:10, 50:50, 50:50, 0:100 and 90:10, with run times of 0.0, 4.0, 9.0, 10.0 and 15.0 min, respectively. Injection volume was set at 50 µL. PLA quantification was calculated according to a standard calibration curve with concentrations ranging between 10 and 1000 mg/L.

#### 4.8.2. T-2 Toxin Extraction and Quantification

After the incubation period, cultures were filtrated with Nalgene™ Rapid-Flow™ Filters of 0.45 μm pore size (Thermofischer Scientific, Waltham, MA, USA) to remove microorganisms. Filtrates were then extracted with 70 mL of ethyl acetate and shaken on a Universal Shaker SM 30 B Control Edmund Bühler^®^ (Thermofischer Scientific, Waltham, MA, USA) set at 150 rpm overnight. The organic phase was recovered and evaporated until dry under a rotavapor set at 60 °C. Samples were resuspended with 2 mL of acetonitrile/water (30/70, v/v) mixture and filtered through 0.45 µm PTFE syringe filters (Sigma Aldrich, St. Quentin Fallavier, France). Samples were conserved at 4 °C until further analysis. T-2 toxin was analyzed by Gemini C18 columns, 150 mm × 4.6 mm, 3 μm and a pre-column with the same characteristics (Phenomenex). As for PLA, T-2 toxin was detected and quantified using HPLC-DAD (Dionex, Sunnyvale, CA, USA) according to the methodology described by Medina et al. [59]. T-2 toxin quantification was calculated according to a standard calibration curve with concentrations ranging between 0.2 and 50 μg/mL.

### 4.9. Statistical Analysis

First, the normal distribution of data was tested by the Shapiro-Wilk test. Then, one-way analysis of variance (ANOVA) followed by Dunnett’s multiple comparisons test was used to analyze the effect of PLA on *F. langsethiae 2297* and *F. sporotrichioides 186* growth and their T-2 production. One-way ANOVA followed by a Tukey’s multiple comparisons test was used to analyze the differences between control and co-culture or sequential culture conditions. The statistical analysis of data was carried out with GraphPad Prism 8 software (GraphPad Software, La Jolla, CA, USA). Differences were considered to be statistically significant when the *p*-value was lower than 0.05. Graphical values are represented by mean ± standard deviation (SD).

## References

[1] The Brewers of Europe (2019). European Beer Trends—Statistics Report.

[2] Laitila A., Hill E.A. (2015). Toxigenic fungi and mycotoxins in the barley-to-beer chain. Food Science, Technology and Nutrition, Brewing Microbiology.

[3] Creppy E.E. (2002). Update of survey, regulation and toxic effects of mycotoxins in Europe. Toxicol. Lett..

[4] Bennett J.W., Klich M. (2013). Mycotoxins. Clin. Microbiol. Rev..

[5] Kirinčič S., Sˇkrjanc B., Kos N., Kozolc B., Pirnat N., Tavčar-Kalcher G. (2015). Mycotoxins in cereals and cereal products in Slovenia—Official control of foods in the years 2008–2012. Food Control.

[6] Pleadin J., Vahčić N., Perši N., Ševelj D., Markov K., Frece J. (2013). *Fusarium* mycotoxins’ occurrence in cereals harvested from Croatian fields. Food Control.

[7] Běláková S., Benešová K., Čáslavský J., Svoboda Z., Mikulíková R. (2014). The occurrence of the selected *fusarium* mycotoxins in czech malting barley. Food Control.

[8] Morcia C., Tumino G., Ghizzoni R., Badeck F.W., Lattanzio V.M.T., Pascale M., Terzi V. (2016). Occurrence of *Fusarium langsethiae* and T-2 and HT-2 toxins in Italian malting barley. Toxins (Basel).

[9] Pascari X., Ramos A.J., Marín S., Sanchís V. (2018). Mycotoxins and beer. Impact of beer production process on mycotoxin contamination. A review. Food Res. Int..

[10] Donnell K.O., Mccormick S.P., Busman M., Proctor R.H., Ward J., Doehring G., Geiser D.M., Alberts J.F., Rheeder J.P., Donnell K.O. (2018). 1984 “Toxigenic *Fusarium* Species: Identity and Mycotoxicology” revisited. Mycologia.

[11] Gilgan M.W., Smalley E.B., Strong F.M. (1966). Isolation and partial characterization of a toxin from *Fusarium tricinctum* on moldy corn. Arch. Biochem. Biophys..

[12] Thrane U., Adler A., Clasen P.E., Galvano F., Langseth W., Lew H., Logrieco A., Nielsen K.F., Ritieni A. (2004). Diversity in metabolite production by *Fusarium langsethiae, Fusarium poae*, and *Fusarium sporotrichioides*. Int. J. Food Microbiol..

[13] van der Fels-Klerx H., Stratakou I. (2010). T-2 toxin and HT-2 toxin in grain and grain-based commodities in Europe: Occurrence, factors affecting occurrence, co-occurrence and toxicological effects. World Mycotoxin J..

[14] IARC (1993). Some naturally occurring substances: Food items and constituents, heterocyclic aromatic amines and mycotoxins. IARC Monogr. Eval. Carcinog. Risks Humans..

[15] European Commission (EC) (2013). Recomendations on the presence of T-2 and HT-2 toxin in cereals and cereal products. Off. J. Eur. Union.

[16] Fandohan P., Gnonlonfin B., Hell K., Marasas W.F.O., Wingfield M.J. (2005). Natural occurrence of *Fusarium* and subsequent fumonisin contamination in preharvest and stored maize in Benin, West Africa. Int. J. Food Microbiol..

[17] Adegoke G.O., Letuma P., Makun H. (2013). Strategies for the Prevention and Reduction of Mycotoxins in Developing Countries. Mycotoxin and Food Safety in Developing Countries.

[18] Magan N., Aldred D. (2007). Post-harvest control strategies: Minimizing mycotoxins in the food chain. Int. J. Food Microbiol..

[19] Agriopoulou S., Stamatelopoulou E., Varzakas T. (2020). Control Strategies: Prevention and Detoxification in Foods. Foods.

[20] Ferrigo D., Raiola A., Causin R. (2016). Fusarium Toxins in Cereals: Occurrence, Legislation, Their Management. Molecules.

[21] Gupta P.K., Aggarwal M. (2018). Toxicity of fungicides. Veterinary Toxicology.

[22] Zubrod J., Bundschuh M., Arts G., Brühl C., Imfeld G., Knäbel A., Payraudeau S., Rasmussen J., Rohr J., Scharmüller A. (2019). Fungicides—An Overlooked Pesticide Class ?. Env. Sci Technol.

[23] Mansfield B.E., Oltean H.N., Oliver B.G., Hoot S.J., Leyde S.E., Hedstrom L., White T.C. (2010). Azole drugs are imported by facilitated diffusion in *Candida albicans* and other pathogenic fungi. PLoS Pathog..

[24] Tano Z.J., Stoytcheva M. (2011). Ecological Effects of Pesticides. Pesticides in the Modern Word—Risk and Benefits.

[25] Price C.L., Parker J.E., Warrilow A.G., Kelly D.E., Kelly S.L. (2015). Azole fungicides—understanding resistance mechanisms in agricultural fungal pathogens. Pest Manag. Sci..

[26] Rahman M.M., Flory E., Koyro H.W., Abideen Z., Schikora A., Suarez C., Schnell S., Cardinale M. (2018). Consistent associations with beneficial bacteria in the seed endosphere of barley (*Hordeum vulgare* L.). Syst. Appl. Microbiol..

[27] Jackson L.S., Katta S.K., Fingerhut D.D., DeVries J.W., Bullerman L.B. (2002). Effects of Baking and Frying on the Fumonisin B 1 Content of Corn-Based Foods. J. Agric. Food Chem..

[28] Kabak B., Dobson A.D.W., Var I. (2006). Strategies to prevent mycotoxin contamination of food and animal feed: A review. Crit. Rev. Food Sci. Nutr..

[29] Terzi V., Tumino G., Stanca A.M., Morcia C. (2014). Reducing the incidence of cereal head infection and mycotoxins in small grain cereal species. J. Cereal Sci..

[30] Strub C., Pocaznoi D., Lebrihi A., Fournier R., Mathieu F. (2010). Influence of barley malting operating parameters on T-2 and HT-2 toxinogenesis of *Fusarium langsethiae*, a worrying contaminant of malting barley in Europe. Food Addit. Contam. Part A Chem. Anal. Control. Expo. Risk Assess..

[31] Mastanjevi K., Krstanovic V., Mastanjevic K., Šarkanj B. (2018). Malting and Brewing Industries Encounter *Fusarium spp*. Related Problems. Toxins (Basel).

[32] Sadiq F.A., Yan B., Tian F., Zhao J., Zhang H., Chen W. (2019). Lactic Acid Bacteria as Antifungal and Anti-Mycotoxigenic Agents: A Comprehensive Review. Compr. Rev. food Sci. food Saf..

[33] Rouse S., van Sinderen D. (2008). Bioprotective Potential of Lactic Acid Bacteria in Malting and Brewing. J. Food Prot..

[34] Geissler A.J., Behr J., Kamp K. (2016). Von Vogel, R.F. Metabolic strategies of beer spoilage lactic acid bacteria in beer. Int. J. Food Microbiol..

[35] Suzuki K. (2011). 125th Anniversary Review: Microbiological Instability of Beer Caused by Spoilage Bacteria. J. Inst. Brew..

[36] Boivin P., Malanda M. (1999). Inoculation by Geotrichum Candidum during Malting of Cereals or Other Plants. US Patent.

[37] Dieuleveux V., Van Der Pyl D., Chataud J., Gueguen M. (1998). Purification and characterization of anti-Listeria compounds produced by *Geotrichum candidum*. Appl. Environ. Microbiol..

[38] Dieuleveux V., Lemarinier S., Guéguen M. (1998). Antimicrobial spectrum and target site of D-3-phenyllactic acid. Int. J. Food Microbiol..

[39] Lucchini J.J., Corre J., Cremieux A. (1990). Antibacterial activity of phenolic compounds and aromatic alcohol. Res. Microbiol..

[40] Gastélum-Martínez E., Compant S., Taillandier P., Mathieu F. (2012). Control of T-2 toxin in *Fusarium langsethiae* and *Geotrichum candidum* co-culture. Arh. Hig. Rada Toksikol..

[41] Torp M., Nirenberg H.I. (2004). *Fusarium langsethiae sp. nov*. on cereals in Europe. Int. J. Food Microbiol..

[42] Imathiu S.M., Edwards S.G., Ray R.V., Back M.A. (2013). *Fusarium langsethiae*—A HT-2 and T-2 Toxins Producer that Needs More Attention. J. Phytopathol..

[43] Foroud N.A., Baines D., Gagkaeva T.Y., Thakor N., Badea A., Steiner B., Bürstmayr M., Bürstmayr H. (2019). Trichothecenes in Cereal Grains – An Update. Toxins (Basel).

[44] Vermeulen N., Gánzle M.G., Vogel R.F. (2006). Influence of peptide supply and cosubstrates on phenylalanine metabolism of *Lactobacillus sanfranciscensis* DSM20451T and *Lactobacillus plantarum* TMW1.468. J. Agric. Food Chem..

[45] Li X., Jiang B., Pan B. (2007). Biotransformation of phenylpyruvic acid to phenyllactic acid by growing and resting cells of a *Lactobacillus* sp.. Biotechnol. Lett..

[46] Mu W., Chen C., Li X., Zhang T., Jiang B. (2009). Optimization of culture medium for the production of phenyllactic acid by *Lactobacillus* sp. SK007. Bioresour. Technol..

[47] Chaudhari S., Gokhale D. (2016). Phenyllactic Acid: A Potential Antimicrobial Compound in Lactic acid Bacteria. J. Bacteriol. Mycol. Open Access.

[48] Corsetti A., Gobbetti M., Rossi J., Damiani P. (1998). Antimould activity of sourdough lactic acid bacteria: Identification of a mixture of organic acids produced by *Lactobacillus sanfrancisco* CB1. Appl. Microbiol. Biotechnol..

[49] Hassan Y.I., Bullerman L.B. (2008). Antifungal activity of *Lactobacillus paracasei ssp. tolerans* isolated from a sourdough bread culture. Int. J. Food Microbiol..

[50] Sathe S.J., Nawani N.N., Dhakephalkar P.K., Kapadnis B.P. (2007). Antifungal lactic acid bacteria with potential to prolong shelf-life of fresh vegetables. J. Appl. Microbiol..

[51] Lavermicocca P., Valerio F., Evidente A., Lazzaroni S., Corsetti A., Gobbetti M. (2000). Purification and characterization of novel antifungal compounds from the sourdough *Lactobacillus plantarum* strain 21B. Appl. Environ. Microbiol..

[52] Lavermicocca P., Valerio F., Visconti A. (2003). Antifungal activity of phenyllactic acid against molds isolated from bakery products. Appl. Environ. Microbiol..

[53] Dieuleveux V., Guéguen M. (1998). Antimicrobial effects of D-3-phenyllactic acid on *Listeria monocytogenes* in TSB-YE medium, milk, and cheese. J. Food Prot..

[54] Morcia C., Tumino G., Ghizzoni R., Bara A., Salhi N., Terzi V. (2017). In Vitro Evaluation of Sub-Lethal Concentrations of Plant-Derived Antifungal Compounds on FUSARIA Growth and Mycotoxin Production. Molecules.

[55] Mateo E.M., Gómez J.V., Gimeno-Adelantado J.V., Romera D., Mateo-Castro R., Jiménez M. (2017). Assessment of azole fungicides as a tool to control growth of *Aspergillus flavus* and aflatoxin B1 and B2 production in maize. Food Addit. Contam. Part A Chem. Anal. Control. Expo. Risk Assess..

[56] Audenaert K., Vanheule A., Höfte M., Haesaert G. (2013). Deoxynivalenol: A Major Player in the Multifaceted Response of *Fusarium* to Its Environment. Toxins (Basel)..

[57] Piegza M., Witkowska D., Stempniewicz R. (2014). Enzymatic and molecular characteristics of *Geotrichum candidum* strains as a starter culture for malting. J. Inst. Brew..

[58] Hattingh M., Alexander A., Meijering I., van Reenen C.A., Dicks L.M.T. (2014). Malting of barley with combinations of *Lactobacillus plantarum, Aspergillus niger, Trichoderma reesei, Rhizopus oligosporus* and *Geotrichum candidum* to enhance malt quality. Int. J. Food Microbiol..

[59] Medina A., Valle-Algarra F.M., Jiménez M., Magan N. (2010). Different sample treatment approaches for the analysis of T-2 and HT-2 toxins from oats-based media. J. Chromatogr. B Anal. Technol. Biomed. Life Sci..

